# Aspirin Reduces Ischemia-Reperfusion Injury Induced Endothelial Cell Damage of Arterial Grafts in a Rodent Model

**DOI:** 10.3390/antiox11020177

**Published:** 2022-01-18

**Authors:** Gábor Veres, Kálmán Benke, Roland Stengl, Yang Bai, Klára Aliz Stark, Alex Ali Sayour, Tamás Radovits, Sivakkanan Loganathan, Sevil Korkmaz-Icöz, Matthias Karck, Gábor Szabó

**Affiliations:** 1 Department of Cardiac Surgery, University of Heidelberg, INF 326, 69120 Heidelberg, Germany; byby23@126.com (Y.B.); starklaraliz@gmail.com (K.A.S.); alexali.sayour@gmail.com (A.A.S.); sivakkanan@gmail.com (S.L.); korkmaz@uni-heidelberg.de (S.K.-I.); matthias.karck@med.uni-heidelberg.de (M.K.); Gabor.Szabo@uk-halle.de (G.S.); 2 Department of Cardiac Surgery, Martin Luther University Halle-Wittenberg, Ernst-Grube Str. 40, 06120 Halle (Saale), Germany; kalman.benke@gmail.com; 3 Heart and Vascular Center, Semmelweis University, Varosmajor u. 68, 1122 Budapest, Hungary; rolandstengl01@gmail.com (R.S.); radovitstamas@yahoo.com (T.R.)

**Keywords:** aspirin, preconditioning, ischemia-reperfusion injury, CABG, arterial graft, patency

## Abstract

Long-term graft patency determines the prognosis of revascularization after coronary artery bypass grafting (CABG). Ischemia-reperfusion (I/R) injury of the graft suffered during harvesting and after implantation might influence graft patency. Aspirin, a nonsteroidal anti-inflammatory drug improves the long-term patency of vein grafts. Whether aspirin has the same effect on arterial grafts is questionable. We aimed to characterize the beneficial effects of aspirin on arterial bypass grafts in a rodent revascularization model. We gave Lewis rats oral pretreatment of either aspirin (*n* = 8) or saline (*n* = 8) for 5 days, then aortic arches were explanted and stored in cold preservation solution. The third group (*n* = 8) was a non-ischemia-reperfusion control. Afterwards the aortic arches were implanted into the abdominal aorta of recipient rats followed by 2 h of reperfusion. Endothelium-dependent vasorelaxation was examined with organ bath experiments. Immunohistochemical staining were carried out. Endothelium-dependent maximal vasorelaxation improved, nitro-oxidative stress and cell apoptosis decreased, and significant endothelial protection was shown in the aspirin preconditioned group, compared to the transplanted control group. Significantly improved endothelial function and reduced I/R injury induced structural damage were observed in free arterial grafts after oral administration of aspirin. Aspirin preconditioning before elective CABG might be beneficial on free arterial graft patency.

## 1. Introduction

Coronary artery bypass grafting (CABG) is the most durable treatment for coronary artery disease (CAD) [1]. However, graft failure especially in the early postoperative period occurs in approximately 5–10% of patients with arterial grafts, and even more frequently in patients with vein grafts [2]. Hence, focus was directed towards pre- and postoperative medications that could have the potential to improve graft patency and reduce cardiovascular events after CABG.

The occlusion of bypass grafts within the first postoperative month is mostly due to thrombosis triggered by surgical trauma [2] and/or ischemia-reperfusion (I/R) injury induced endothelial dysfunction. Recent studies showed that the degree of I/R injury might be also one of the major determinants among several factors influencing the long-term patency [3,4,5,6].

Acetylsalicylic acid (ASA) is a traditional non-steroid anti-inflammatory drug with antipyretic, analgesic, antithrombotic and anti-inflammatory effects. Aspirin achieves platelet inhibition by blocking the cyclooxygenase-1 enzyme, thereby suppressing the production of thromboxane, a powerful platelet agonist and vasoconstrictor. Nevertheless, ASA is also a potent anti-inflammatory drug by inhibiting the cyclooxigenases (COX) [7]. 

Preoperative aspirin treatment has been shown to reduce the risk of early vein graft occlusion [8], and to improve short- and long-term outcomes after CABG [9,10]. However, it is less established whether this beneficial effect of pre-CABG aspirin also applies to arterial grafts. A former study was carried out on preoperative aspirin use in patients undergoing CABG and a trend towards improved early patency of Y-grafts of internal mammary artery with aspirin use was shown; however, statistical significance was not reached [11].

To study the potential short-term benefits of the pharmacological pretreatment with aspirin on arterial grafts, we used a well-established in vivo model of arterial revascularization [12,13,14]. Based on the pathomechanism of I/R injury and endothelial dysfunction after CABG and on the mechanism of action of aspirin, we hypothesize that aspirin pretreatment can reduce I/R injury and endothelial damage in the applied experimental model of revascularization.

## 2. Materials and Methods

### 2.1. Ethical Statement

The experimental study was reviewed and approved by the Ethical Committee for Animal Experimentation (Karlsruhe, Baden-Württemberg, Germany; protocol code: 35-9185.81/G-33/18).

### 2.2. Animals

**Figure 1:**
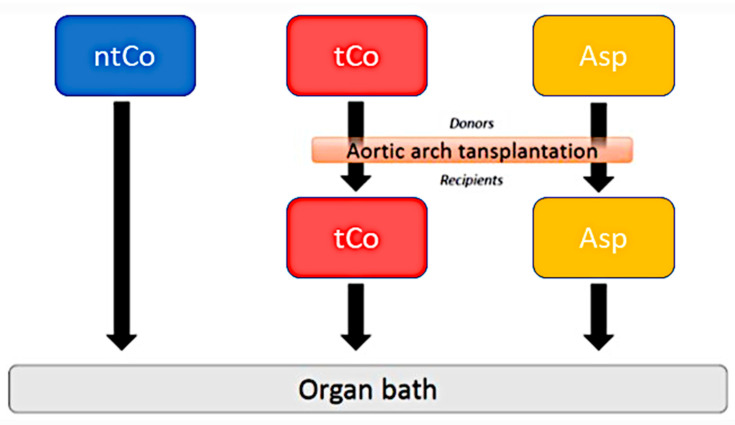
The applied experimental design. tCo: transplanted control group; Asp: Aspirin group; ntCo: non-transplanted control group.

Lewis rats (weight: 250–350 g; male, Charles River Laboratories, Sulzfeld, Germany) were randomly divided into three groups: (1) transplanted control group (tCo, *n* = 8): Lewis rats were given a polyethylene glycol vehicle, (2) Aspirin group (Asp, *n* = 8): Lewis rats were treated with ASA. ASA was prepared as a suspension in a polyethylene glycol vehicle at a volume of 2 mL/kg. A dose of 115 mg/kg was administered once daily, by oral gavage, for 5 days. The results of previous experiments were used to determine the application and dosage of ASA [8]. The third (3) group served as a non-transplanted control (ntCo, *n* = 8). The experimental design is demonstrated in Figure 1. Procedures concerning animals conformed to the Guide for the Care and Use of Laboratory Animals [15]. 

### 2.3. Aortic Transplantation

The experimental model was established according to the reported method [12,13,14]. Lewis rats were anesthetized with Isofluran (3% to initiate anaesthesia, 1.75–2.5% to maintain anaesthesia). In addition, subcutaneous buprenorphine was used (0.05–0.1 mg/kg) 45 min before surgery. For excision of the aortic grafts, the rats were anesthetized again with Isofluran. 

The aortic arch of donor rats was excised and flushed with cold physiological saline solution, followed by an 80-min-long storage in cold saline. At the end of this ischemic period, heterotopical transplantation of the arterial graft with two end-to-end anastomoses to the recipient’s abdominal aorta was carried out (~40 min warm ischemia). After 120 min, an overdose of intraperitoneal sodium pentobarbital (150 mg/kg) was applied to sacrifice the rats with the transplanted arterial graft. Aortic graft segment was excised from the abdomen, sliced into 4-mm-wide rings that were mounted in organ baths (Radnoti Glass Technology, Monrovia, CA, USA), as also reported before [14].

The segments of the implanted aortic graft (after 2 h reperfusion) were fixed in buffered paraformaldehyde solution (4%) and embedded in paraffin. After that, 4-μm-thick sections were placed on adhesive slides, as described before [14]. 

### 2.4. In Vitro Organ Bath Experiments

Functional vascular measurements were carried out on the excised aortic rings, as previously reported [16]. Briefly, 2 aortic rings/animal in each group were mounted on stainless steel hooks under 2 g tension, followed by 60-min-long equilibration [16]. Before every investigation, the rings were prepared for stable contractions with potassium chloride (KCl, 80 mM). Phenylephrine (PE, 10^− 6^ M) was used to rinse and preconstrict the aortic rings until reaching a stable plateau, and cumulative concentrations of acetylcholine (ACh, 10^− 9^–10^− 4^ M) were added to assess the relaxation responses. The response of smooth muscle cells was also tested with sodium nitroprusside (SNP, 10^− 10^–10^− 5^ M). In addition, the sensitivity of the aortic rings to vasorelaxants (pD2) was investigated. Half-maximum response (EC50) values were obtained from individual concentration-responses by fitting experimental data to a sigmoidal equation with the use of Origin 7.0 (Microcal Software, Northampton, MA, USA).

### 2.5. Immunohistochemical Staining (TUNEL, CD-31, Nitrotyrosine, cGMP, Caspase-3, eNOS, VCAM-1)

We performed terminal deoxynucleotidyl transferase dUTP nick end labeling (TUNEL), cluster of differentiation 31 (CD-31), nitrotyrosine, Caspase-3 and vascular cell adhesion molecule 1 (VCAM-1) immunohistochemical staining as reported before [16,17]. 

The brief description of TUNEL staining: 50 µL of Terminal deoxynucleotidyl Transferase (TdT) enzyme and TUNEL Reaction mixture were used to incubate the sections for 1 h at 37 °C in the dark, followed by washing with phosphate-buffered saline (PBS). The slides were mounted using 4′,6-diamidino-2-phenylindole (DAPI)-Fluoromount-G™ (SouthernBiotech, Birmingham, AL, USA), covered with cover glass and analysed under a fluorescence microscope. 

CD-31 staining: As previously described [14], anti-CD31 mouse IgG (Santa Cruz Biotechnology Inc, Heidelberg, Germany) was applied following the manufacturer’s instructions. Microscopic examination of endothelium covered areas of aortic arches was carried out. Additional staining: briefly, the slices were immersed with (a) xylene, three 5-min-long washes, (b) 100% ethanol, two 10-min-long washes, (c) 95% ethanol, two 10-min-long washes, (d) 70% ethanol, two 10- minute-long washes, (e) 50% ethanol, two 10-min-long washes, (f) deionized water, two 5-min-long washes. The tissue sections were quenched with 3.0% hydrogen peroxide in PBS for 20 min to block the endogenous peroxidase activity. Afterwards, the sections were washed by immersing them in distilled water for 5 min. Then the slides were placed in the staining dish with citrate buffer, and put in the microwave (700 watt, 20 min) to achieve antigen retrieval. After that, we put the staining dish on the lab bench for 20–30 min before carrying out the staining. A circle on the slide around the tissue was drawn with a hydrophobic barrier pen. Any non-specific binding was blocked by the incubation of the tissue sections with 2% horse serum in PBS for 30 min. Afterwards, all the blocking serum was removed. Then the primary antibody (VCAM-1, caspase-3 (Novus Biologicals, Littleton, CO, USA), nitrotyrosine (MilliporeSigma, Burlington, MA, USA)) which was diluted in 2% horse serum in PBS were added, followed by a 2-h-long incubation. After that, we washed each section twice with PBS for 10 min. 

Afterwards, a biotin conjugated secondary antibody was added and incubated for 30 min. After that, the sections underwent washing with PBS twice for 10 min each. Then, avidin-biotin complex-horseradish peroxidase (ABC-HRP) reagent (VECTASTAIN universal elite ABC kit, Burlingame, CA, USA) was added, followed by a 30-min-long incubation. After that 3, 3-diaminobenzidine (DAB, VECTOR DAB kit, Burlingame, CA, USA) was applied to visualize the expression of target protein. The reaction in which the chromogenic reaction turned the epitope sites brown was monitored. Each section was then washed in PBS twice for 10 min. Haematoxylin was used to counterstain nuclei following the manufacturer’s protocol. Tissue sections were dehydrated by moving slides through the following solutions twice for 2 min each: (a) 95% ethanol (b) 100% ethanol (c) xylene. At the end, mounting media to slides and top with coverslips were added.

### 2.6. Drugs

Aspirin was bought from Bayer HealthCare (Wuppertal, Germany). PE, ACh and SNP were bought from Sigma-Aldrich (Steinheim, Germany). 

### 2.7. Statistical Analysis

All data are expressed as means ± standard error of mean (SEM). Data were tested for normal distribution (Shapiro–Wilk test) and where they met the requirements for parametric analysis, means were tested by one-way analysis of variance followed by a Student *t* test with Bonferroni correction for multiple comparisons. A value of p less than 0.05 was considered statistically significant. SPSS Statistics 24 (IBM Corp, Armonk, NY, USA) software was used for data analysis and visualization.

## 3. Results

### 3.1. Vascular Function of Aortic Rings

**Figure 2:**
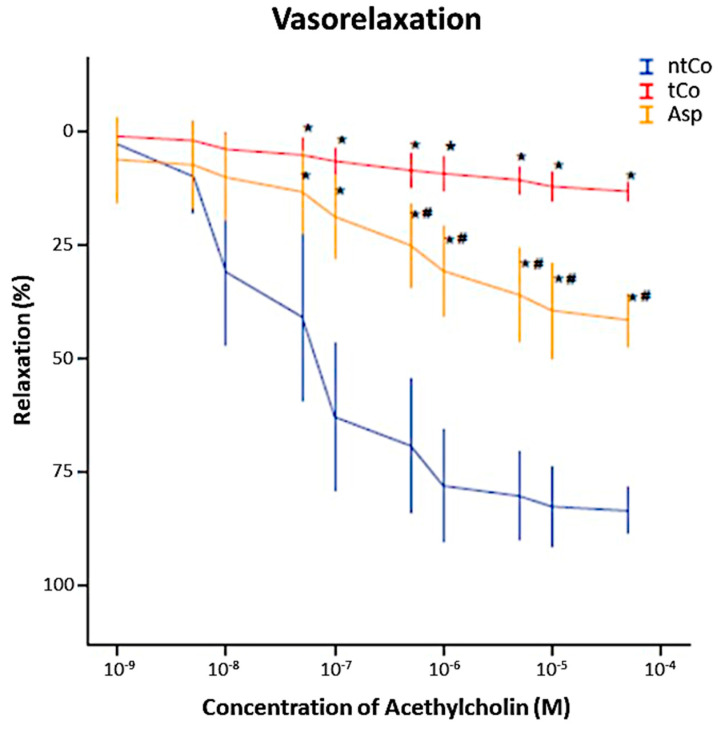
Result of the organ bath functional measurement. Aspirin pretreatment resulted in a significantly better graft function compared to the tCo group. * *p* < 0.05 vs. ntCo, # *p* < 0.05 vs. tCo.

ACh induced a concentration-dependent relaxation in PE precontracted arterial rings (Figure 2). After reperfusion, the maximum endothelium-dependent vasorelaxation (Rmax) to ACh was significantly decreased in the tCo (14 ± 2%) and Asp (42 ± 4%) groups as compared to the ntCo (84 ± 3%), showing endothelial function impairment (*p* < 0.05). However, pretreatment with ASA significantly improved the endothelium-dependent function of arterial rings when compared to the tCo group (*p* < 0.05) (Figure 2). There was no significant difference in Rmax for the vasorelaxation to SNP between the groups. PE induced concentration-dependent contraction of the rings in all groups. The value of maximum contraction for PE is higher in the tCo and Asp groups than in the ntCo group.

### 3.2. The Effect of Ischemia-Reperfusion Injury on Arterial Graft (Rate of Oxidative Stress and Apoptosis)

**Figure 3:**
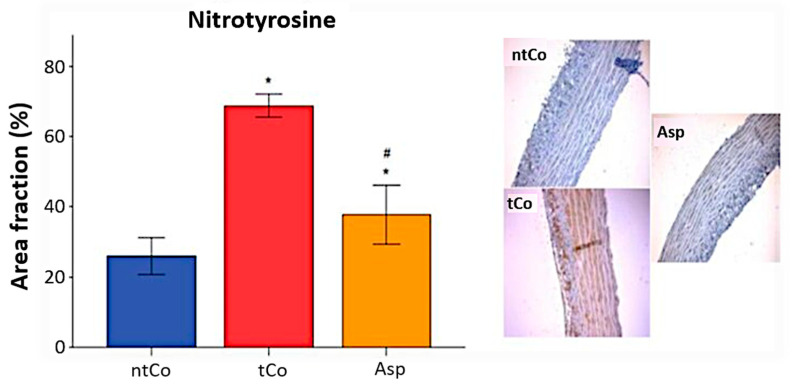
Aspirin significantly decreased the nitro-oxidative stress caused by ischemia- reperfusion injury, which resulted in decreased NT-3 positivity. * *p* < 0.05 vs. ntCo, # *p* < 0.05 vs. tCo. Magnification: 200×.

The level of oxidative stress in the vascular wall of the arterial graft was assessed by nitrotyrosine immunoreactivity, characterized by nitrotyrosine-3 positive area. The intensity of nitrotyrosine staining in the tCo (69 ± 1%) and Asp (38 ± 3%) groups was enhanced compared to the ntCo (26 ± 2%) (*p* < 0.05), but Asp significantly decreased the level of nitro-oxidative stress compared to the tCo (*p* < 0.05) (Figure 3).

**Figure 4:**
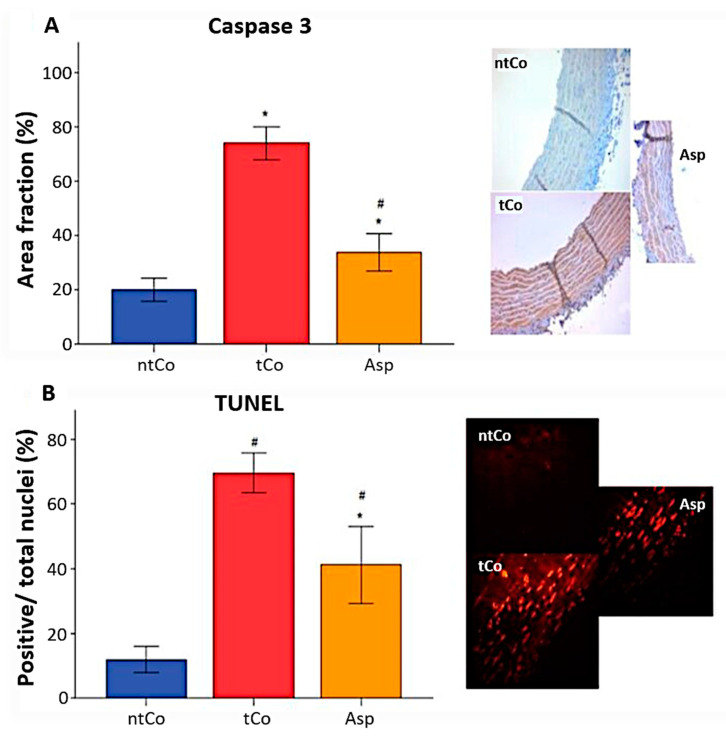
Antiapoptotic effect of the Aspirin pretreatment was found based on (**A**) the significantly increased Caspase-3 immunoreactivity and (**B**) the significantly increased TUNEL positivity. * *p* < 0.05 vs. ntCo, # *p* < 0.05 vs. tCo. Magnification: 200×.

A higher number of caspase-3 positive cells, measured as caspase-3 positive area, was observed in the tCo (74 ± 3%) and Asp (34 ± 3%) groups indicating more severe apoptosis as opposed to the ntCo (20 ± 2%) (*p* < 0.05). ASA pretreatment significantly reduced apoptosis rate compared to the tCo group (*p* < 0.05) (Figure 4A). In addition, the intensity of TUNEL-positive area of the arterial graft in the tCo (70 ± 3%) and Asp (41 ± 5%) groups was increased, indicating a higher level of DNA-fragmentation in comparison to the ntCo (12 ± 2%) (*p* < 0.05). However, significantly decreased DNA strand breaks were observed in the Asp group in comparison with the tCo (Figure 4B).

We performed immunohistochemical staining for eNOS to identify the amount of eNOS in the wall of arterial graft. An enhanced reactivity for eNOS was observed in the arterial rings of the tCo and Asp groups, which was significantly lower in the ntCo group.

**Figure 5:**
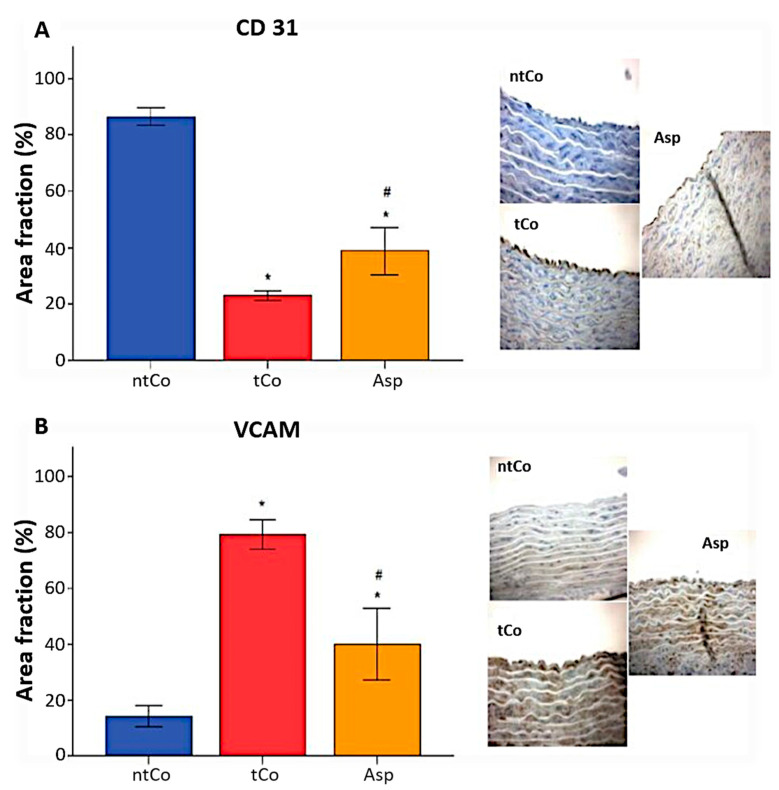
We carried out histomorphological analysis of the endothelium by identifying uninjured and activated endothelial cells. (**A**) CD-31 positive area, reflecting the uninjured endothelium surface was significantly more extensive in the Asp group compared to the tCo group. (**B**) VCAM immunoreactivity was increased after transplantation, which leads to increased leukocyte invasion. Aspirin pretreatment decreased the VCAM positivity of the endothelium. * *p* < 0.05 vs. ntCo, # *p* < 0.05 vs. tCo. Magnification: 200×.

The inner walls of the aortic segment in the non-transplanted control group showed 87 ± 1% CD-31 positive endothelium area, demonstrating a high area of uninjured endothelium, whereas it was significantly decreased in the tCo (23 ± 1%) and Asp (39 ± 3%) groups (*p* < 0.05). The CD-31 positive reaction was significantly higher in the Asp group than in the tCo group (*p* < 0.05) (Figure 5A). Immunohistochemical staining revealed a high VCAM-1 positive endothelium area, which leads to a more pronounced leukocyte invasion in the tCo (79 ± 2%) and Asp groups (40 ± 5%), as opposed to the ntCo (14 ± 2%) (*p* < 0.05), however, VCAM-1 scores decreased significantly in the Asp group compared to the tCo group (*p* < 0.05) (Figure 5B).

## 4. Discussion

In this study, the benefits of the pretreatment with aspirin on free arterial grafts were assessed in an in vivo arterial revascularization model. In accordance with the literature, ischemia and reperfusion resulted in a decline in the endothelial function of free arterial grafts [4,18]. However, we have shown that pretreatment with aspirin had beneficial effects on the arterial grafts when compared to the transplanted control group. Importantly, preoperative usage of aspirin significantly improved the endothelial function of the free arterial grafts and reduced the apoptosis rate. Pretreatment with aspirin also decreased the important ischemia-reperfusion injury determinant nitro-oxidative stress [19]. The adhesion molecule VCAM-1 plays a role in leukocyte adhesion and extravasation between endothelial cells [20], and the interaction between endothelial cells and leukocytes, and an increased reactive oxygen species production by the leukocytes result in endothelial dysfunction [21]. VCAM-1 positivity was significantly decreased, while the CD31 positive area, indicating uninjured endothelium was significantly enhanced in case of aspirin pretreatment. As endothelial function is one of the major determinants of early- and late-term vascular graft patency after cardiac surgery [12], these findings provide new insights in the endothelium protection of free arterial grafts, which could lead to improved patency rates of these type of grafts.

Although aspirin has no direct effect on vascular function, it provides beneficial protection against many pathological conditions such as atherosclerosis [22] and myocardial infarction [23]. Pre- and postoperative use of aspirin during CABG improves early patency rates and reduces thrombotic events in saphenous vein grafts [8,24]. However, these findings cannot be extrapolated to arterial grafts, as there are marked differences in endothelial function between arterial and venous grafts [18]. The effect of pre-CABG aspirin use on arterial grafts is less established and debatable. 

The preoperative preconditioning with platelet inhibitors were widely examined throughout the years. Internal mammary artery (IMA) and vein grafts were found to have excellent patency rates at 1 year, but aspirin did not result in better patency compared to placebo, and no patency difference was observed between IMA and vein grafts to the LAD, when patients were given aspirin or placebo before or after CABG surgery [25]. Another study on preoperative use in patients undergoing CABG showed a trend towards improved early patency of Y grafts and IMA with aspirin use, however, it did not reach statistical difference [11].

In another clinical study, the efficacy and safety of aspirin, aspirin plus dipyridamole, and oral anticoagulant agents were compared in the prevention of internal mammary artery graft occlusion. As concluded, aspirin plus dipyridamole or oral anticoagulant agents did not improve internal mammary artery graft patency at 1 year when compared to low dose aspirin alone. However, there is evidence for an increased overall clinical event rate if dipyridamole is added to aspirin [26]. Goldman et al. investigated the patency rate of saphenous vein- and left internal mammary artery grafts 10 years after CABG, and the postoperative use of aspirin was found to be a positive significant predictor of graft patency [27]. It was also revealed that the administration of aspirin within 48 h after CABG significantly reduced the incidence of myocardial infarction, stroke, renal failure, bowel infarction and mortality, while the risk of bleeding, infection, gastritis and impaired wound healing did not increase [28]. Aspirin administration 5 days prior to coronary artery bypass surgery was also associated with lower in-hospital mortality, and proved to be safe without the increased risk of reoperation for bleeding or need for blood product transfusion [29]. Due to the significant improvement in saphenous graft patency and reduced morbidity after CABG, the 2011 American College of Cardiology Foundation/American Heart Association Guidelines for Coronary Artery Bypass Surgery recommends the continuation or administration of aspirin preoperatively or 6 h after the operation [30]. These findings demonstrate the benefits and safety of preoperative aspirin use, and our results provide further justification for pretreatment with aspirin with the potential of improving free arterial graft patency. 

No study was conducted about the effect of aspirin pretreatment on the patency of IMA used as free graft. It has been previously reported that the patency of free IMA grafts is 10% to 15% lower than that of an in situ IMA [31]. In addition, our group previously demonstrated a dramatically reduced endothelial function (reduced endothelial-dependent vasodilatation, impaired NO production etc.) of the free arterial graft after short/mid-term reperfusion in various experimental models [32,33].

According to our results, preoperative aspirin could reduce the ischemia-reperfusion injury suffered, when IMA/radial artery is used as free grafts and might influence the early/long-term patency rate, which is crucial for patients with coronary artery disease. Clinical practice is shifting towards continuing aspirin before CABG [34].

Our study has limitations. Rat aortic tissue differs from human arterial grafts (IMA/radial artery) in the structure of the vessel wall, which limits the transferability of the results. However, in the above discussed studies that were carried out on humans, aspirin showed promising results in improving the patency of arterial grafts, which is consistent with our findings, indicating that our results could have a good chance of transferability. As these studies did not involve free arterial grafts, our results warrant the need for investigating the effect of aspirin pretreatment on the patency rate of free arterial grafts in humans.

## 5. Conclusions

To conclude, orally administered aspirin showed a marked improvement on the endothelial function and reduced structural damage caused by I/R injury in free arterial grafts in our experimental setup. ASA preconditioning before elective CABG surgery might be beneficial on the patency of free arterial grafts.

## Data Availability

The data are contained within the article.
